# Artificial Intelligence Has Varied Diagnostic and Predictive Performance in Diagnosing Patellofemoral Osteoarthritis, Trochlear Dysplasia, and Patellofemoral Tracking Abnormalities: A Systematic Review

**DOI:** 10.1016/j.asmr.2025.101269

**Published:** 2025-09-22

**Authors:** Jack Twomey-Kozak, Mikhail A. Bethell, Zoe Wiatt Hinton, Samuel Lorentz, Lucy Meyer, Alex Meyer, Eoghan Hurley, Damon V. Briggs, Kendall Bradley, Jocelyn Wittstein, Brian Lau

**Affiliations:** Department of Orthopaedic Surgery, Duke University, Durham, North Carolina, U.S.A.

## Abstract

**Purpose:**

To systematically review and evaluate the diagnostic efficacy and predictive power of artificial intelligence (AI) models in detecting patellofemoral (PF) compartment pathology and to compare their performance against ground-truth human clinical experts when applicable.

**Methods:**

In accordance with the Preferred Reporting Items for Systematic Reviews and Meta-analyses guidelines, the PubMed, Ovid/MEDLINE, and Cochrane Library databases were searched from inception through May 2024 for studies on AI methods for diagnosing trochlear dysplasia, PF osteoarthritis, or PF instability and tracking abnormalities on cross-sectional imaging. AI model choice, knee pathology, input/output data, performance metrics (accuracy, area under the curve [AUC], precision-recall curve average precision, sensitivity, specificity, positive predictive value, and negative predictive value), sample sizes of datasets, image modalities, and limitations were recorded.

**Results:**

Of 68 studies screened, 17 met the inclusion criteria. Ten studies investigated AI diagnostics for PF osteoarthritis; four, PF tracking and/or instability; and three, trochlear dysplasia. Various deep learning architectures and machine learning algorithms were used. Input data included computed tomography scans, magnetic resonance imaging scans, and radiographs. Output data included anatomic landmark identification and diagnostic predictions. AUC values ranged from 0.664 to 0.990, and accuracy ranged from 74% to 99%. Model performance was moderate to excellent, with AI models consistently surpassing traditional methods in processing times. Common limitations included small sample size, single-center datasets, limited generalizability, and bias due to imbalanced datasets.

**Conclusions:**

AI models showed variable diagnostic performance in identifying PF pathologies and predicting disease progression, with reported AUCs ranging from 0.664 to 0.990 and accuracies between 74% and 99%. Although some studies suggested that AI outperformed traditional diagnostic methods such as interpretation by musculoskeletal radiologists, manual segmentation, or arthroscopy, the degree of superiority was inconsistent and influenced by significant heterogeneity in model architectures, imaging modalities, and reference standards. Given the broad scope of this review and variability across studies, caution is warranted in interpreting these findings, and specific clinical recommendations cannot be made at this time.

**Clinical Relevance:**

AI-based diagnostic tools show promise in supporting the evaluation of PF joint pathologies by potentially improving efficiency and consistency in image interpretation. However, because of the heterogeneity in current models and study designs, the clinical applicability of these tools remains limited. Further refinement and external validation of AI algorithms are needed before their integration into routine clinical decision making can be fully endorsed.

Artificial intelligence (AI), particularly through the advancements of deep learning (DL) and machine learning (ML), has impacted numerous sectors, including orthopaedic surgery.^1^, ^2^, ^3^, ^4^ ML, a subset of AI, involves the development of algorithms that allow computers to learn from and make decisions based on data. DL, a more advanced subset of ML, uses neural networks with many layers to analyze complex patterns and features in large datasets.^5^ The evolution of these technologies has enabled DL algorithms to interpret intricate data patterns, whereas ML has enhanced predictive modeling capabilities. In orthopaedic surgery, AI has been proposed across various stages, from preoperative planning and intraoperative guidance to postoperative rehabilitation. Specifically, there has been an increased emphasis on the use of AI for automated image processing and analysis, which has significantly improved the efficiency of diagnostic processes. Within the field of orthopaedics, AI algorithms are being evaluated to aid clinicians in real-time fracture recognition,^6^, ^7^, ^8^ prognostication of tumor survivorship,^7^^,^^9^, ^10^, ^11^ and postoperative assessment of implant positioning^12^, ^13^, ^14^, ^15^, ^16^ and, more recently, the detection of soft-tissue knee injuries.^17^, ^18^, ^19^, ^20^ To this point, the diagnostic potential of AI is particularly promising because it allows for detection and classification of musculoskeletal (MSK) abnormalities in imaging studies with superior speed compared with traditional ground-truth methods.

The impact of AI is especially relevant in the management of patellofemoral (PF) pathology, in which accurate assessment, diagnostic capability, and predictive modeling of outcomes can be crucial for effective and efficient treatment. Currently, the primary applications of AI involve using magnetic resonance imaging (MRI) and computed tomography (CT) images to detect subtle changes in cartilage, bone, and soft tissues that are indicative of disorders such as PF pain syndrome, chondromalacia patellae, osteoarthritis (OA), and patellar instability.^21^, ^22^, ^23^, ^24^, ^25^ ML models can predict the status of these conditions and the outcomes of various treatment modalities, aiding in the development of personalized treatment plans. For example, AI can assist in identifying patients who are likely to benefit from nonsurgical treatments versus those who may require surgical intervention, thereby optimizing clinical decision making. Furthermore, predictive models based on ML can assess image-based and clinically based patient-specific risk factors to forecast surgical outcomes.^26^^,^^27^

Despite AI’s demonstrated benefits, its application in using radiographic and cross-sectional imaging for diagnosing knee injuries, such as PF OA, trochlear dysplasia, and chondromalacia patellae, as well as PF tracking abnormalities, remains poorly understood. Therefore, the purpose of this study was to systematically review and evaluate the diagnostic efficacy and predictive power of AI models in detecting PF compartment pathology and to compare their performance against ground-truth human clinical experts when applicable. The hypothesis was that AI models would exhibit excellent performance characteristics in the identification and evaluation of PF pathology.

## Methods

### Study Selection

Two independent authors (J.T-K., M.A.B.) completed a query of the literature in accordance with the Preferred Reporting Items for Systematic Reviews and Meta-analyses (PRISMA) guidelines^28^ and reviewed the search results, with each author blinded to the other’s results; a third author (E.H.) was available for arbitration on potential disagreements or discrepancies. Studies were deemed eligible for full-text review based on an initial approval screening of article titles and abstracts.

### Search Criteria and Strategy

A systematic review was performed in accordance with the PRISMA guidelines using the PubMed, Ovid/MEDLINE, and Cochrane Library databases from inception through May 2024. A Boolean search syntax was used to capture the maximum number of articles for screening in the initial search: (("Trochlea" OR "Patellofemoral" OR "Patellofemoral Instability" OR "Trochlear Dysplasia" OR "Knee Disorders" OR "Knee Abnormalities") AND (artificial intelligence OR neural network∗ OR deep learning OR machine learning OR machine intelligence) AND (diagnostic performance OR diagnostic accuracy OR sensitivity OR specificity OR ROC curve OR area under the curve OR AUC OR predictive value of test OR score OR scores OR scoring system OR scoring systems OR observ∗ OR observer variation OR detect∗ OR evaluat∗ OR analy∗ OR assess∗ OR measure∗)).

### Eligibility Criteria

**Fig 1:**
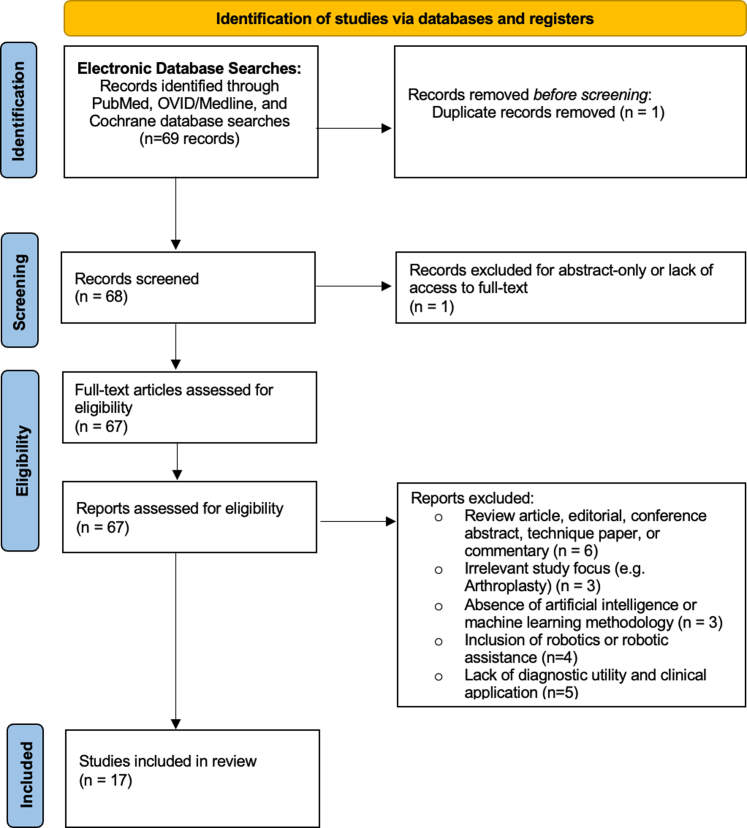
Preferred Reporting Items for Systematic Reviews and Meta-analyses (PRISMA) study selection flow diagram.

Rigorous inclusion criteria were established to ensure the integrity and relevance of the selected literature. Articles were deemed eligible if they met 3 key criteria: The study investigated the development or application of AI specifically for detecting trochlear dysplasia or abnormalities in PF tracking using cross-sectional imaging techniques; the study was published in a peer-reviewed journal in the English language, and the full text of the study was available. Exclusion criteria included articles consisting solely of abstracts, technical papers, cadaveric or animal experiments, or letters to the editor (Fig 1). Finally, the bibliographies of all included studies were cross-referenced to ensure no relevant studies were overlooked.

### Data Extraction

Full-text examination of articles passing the screening process was only undertaken after application of our strict inclusion and exclusion criteria. Furthermore, to ensure completeness, all references cited in the included studies were exhaustively reviewed. Two independent authors (J.T-K., M.A.B.) systematically compiled all pertinent data using a predefined Microsoft Excel data sheet (Microsoft, Redmond, WA) with a modified information extraction table. The columns for these extraction tables included the following: publication data; study title and design; study methodology; knee pathology and anatomic region; sample size (patients); dataset size; AI model; image and model input and output; ground truth; training set, validation set, and test set sizes; performance grading; accuracy grading; area under the curve (AUC) for the receiver operating characteristic curve; conclusions; and limitations.

### Outcomes Analyzed and Statistical Methods

All data were qualitatively synthesized and reported in both narrative fashion and individual table formats. Data extracted were presented as means, medians, ranges, and confidence intervals as appropriate and as provided in respective studies. Outcome measures of interest included accuracy, AUC, average precision (AP), dispersion of data (mean absolute error [MAE], mean absolute deviation [MAD], and/or root-mean-square [RMS]), inter-rater reliability (κ value or intraclass correlation coefficient [ICC]), sensitivity, specificity, positive predictive value (PPV), negative predictive value (NPV), and Dice coefficient. No regression modeling or predictive analytics were performed because the analysis was descriptive in nature and did not require inferential modeling. This absence is disclosed in accordance with reporting considerations for AI-related methodologies. All statistical analyses were performed using R (version 4.0.2, The R Foundation for Statistical Computing, Vienna, Austria), with pooled analysis for quantitative statistical analysis. *P* < .05 was considered statistically significant.

## Results

A total of 69 studies were initially identified through the electronic database search. After the removal of duplicate records, the remaining articles were assessed according to predefined inclusion and exclusion criteria. After a thorough evaluation of full-text eligibility, 17 studies were ultimately selected for inclusion in this review for both quantitative and qualitative data analyses (Fig 1). All studies were classified as Level III and IV evidence, with an average Methodological Index for Non-randomized Studies (MINORS) score of 5.35 ± 0.68. Most of the studies (14 of 17, 82.4%) were retrospective in nature, and 64.7% (11 of 17) used predictive designs. Details of study characteristics and knee pathology of interest are provided in Table 1.

**Table 1:** Study Characteristics and Methodologic Quality Strength Assessment

Study	Knees (Patients), n	Pathology	Prospective or Retrospective	Predictive or Diagnostic	LOE	MINORS Score
Liu et al.^29^ (2023)	14,652 (483)	PF OA	Retrospective	Predictive	IV	5
Bayramoglu et al.^30^ (2022)	5,507	PF OA	Retrospective	Diagnostic	IV	5
Hu et al.^31^ (2022)	104	PF OA or cartilage injury	Prospective	Predictive	III	4
Xu et al.^32^ (2023)	464	Trochlear dysplasia	Retrospective	Predictive	IV	6
Shi et al.^33^ (2021)	41	PF pain syndrome	Retrospective	Diagnostic	IV	6
Yurova et al.^34^ (2024)	15	PF OA	Retrospective	Diagnostic	IV	5
Tuya et al.^35^ (2023)	1,280	PF OA	Retrospective	Diagnostic	IV	6
Tuya et al.^36^ (2023)	1,230	PF maltracking	Retrospective	Predictive	IV	6
Bayramoglu et al.^22^ (2021)	18,436 (2,803)	PF OA	Retrospective	Predictive	IV	5
Hu et al.^37^ (2023)	364 (182)	PF OA	Prospective	Predictive	III	5
Bayramoglu et al.^38^ (2024)	3,276 (1,832)	PF OA	Prospective	Predictive	III	5
Nagawa et al.^39^ (2024)	51 (49)	PF instability	Retrospective	Predictive	IV	5
Barbosa et al.^40^ (2024)	140 (95)	Trochlear dysplasia	Retrospective	Predictive	IV	6
Cerveri et al.^41^ (2018)		Trochlear dysplasia	Retrospective	Predictive	IV	4
Pedoia et al.^42^ (2019)	1,481 (302)	PF OA or cartilage injury	Retrospective	Predictive	IV	6
Cheng et al.^43^ (2020)	176 (93)	PF pain syndrome or OA	Retrospective	Diagnostic	IV	6
Hu et al.^44^ (2024)	600	PF OA	Retrospective	Diagnostic	IV	5

LOE, level of evidence; MINORS, Methodological Index for Non-randomized Studies; OA, osteoarthritis; PF, patellofemoral.

### AI Model Choice

A comprehensive overview of the various AI models used in the included studies, detailing their respective image input types, image planes, and ground truth/reference standards, is presented in Table 2. For a clear explanation of DL concepts, it is important to note that deep neural networks can suffer from the *vanishing gradient problem*, in which gradients become increasingly small as they propagate backward through many layers. This impairs the training of early network layers, making it difficult for the model to learn because important signals become weaker as they move backward through the layers. *Skip connections*, introduced in certain architectures such as residual networks (ResNets), help mitigate this issue by creating direct pathways or shortcuts between nonadjacent layers, allowing gradients to flow more effectively and enabling the training of much deeper networks.

**Table 2:** Overview of AI Model Parameters and Methodology for PF Pathology Studies

Study	AI Model	Image Input	Image Plane	Ground Truth/Reference Standard	Training Set	Validation Set	Testing Set	Model Output
Liu et al.^29^ (2023)	ResNet	CT	Axial	MSK radiologist	Not specified	Not specified	Not specified	Landmark prediction coordinates
Bayramoglu et al.^30^ (2022)	GBM-CNN	Radiography	Sagittal	Comparison of multiple algorithms (GBM)	Not specified	Not specified	Not specified	OARSI and KL grades
Hu et al.^31^ (2022)	MWRN	MRI	Sagittal, coronal, and axial	Arthroscopy	Not specified	Not specified	Not specified	Prediction of image reconstruction
Xu et al.^32^ (2023)	U-Net CNN	MRI	Axial	Radiologist and senior surgeons with >10 yr of experience	370	Not specified	94	Pixel-level regression prediction
Shi et al.^33^ (2021)	MI-CNN	Radiography	Dynamic	Single-input CNN	70%	Not specified	30%	Classification of PFPS
Yurova et al.^34^ (2024)	U-Net CNN	MRI and CT	Sagittal, coronal, and axial	Previous algorithm segmentations	75%	Not specified	25%	Creation of biomechanical model for patellar motion
Tuya et al.^35^ (2023)	HRNet	Radiography	Axial	2 MSK radiologists	1,280	187	129	KL classification of PF OA
Tuya et al.^36^ (2023)	U-Net CNN	Radiography	Sunrise	3 MSK radiologists	1,230	Not specified	201	Prediction of landmarks
Bayramoglu et al.^22^ (2021)	R-CNN	Radiography	Sagittal	2 Independent expert OARSI graders	596	5-Fold cross		Prediction of PF OA status
Hu et al.^37^ (2023)	D-CNN	MRI	Sagittal, coronal, axial, and 3D reconstruction	Biomarker Consortium Database	Not specified	5-Fold cross	Not specified	Prediction of PF OA
Bayramoglu et al.^38^ (2024)	D-CNN	Radiography	Sagittal	2 Independent radiologists	Not specified	5-Fold cross	Not specified	Prediction of PF OA
Nagawa et al.^39^ (2024)	SVM	MRI	Sagittal, coronal, and axial	2 Radiologists with >5 yr of experience	Not specified	5-Fold cross	Not specified	Prediction of PFI
Barbosa et al.^40^ (2024)	U-Net CNN	MRI	Sagittal, coronal, axial, and 3D reconstruction	Expert MSK radiologist	80%	20%	Not specified	Landmark prediction (6, 3, and 7 output channels)
Cerveri et al.^41^ (2018)	SSPA-NN–SSM	CT	Sagittal, coronal, axial, and 3D reconstruction	Previous algorithm segmentations	66	15	Not specified	Prediction of clinical conditions (3 outputs)
Pedoia et al.^42^ (2019)	U-Net CNN	MRI	Coronal	5 Radiologists with >5 yr of experience	65%	20%	15%	Prediction of cartilage lesions (2-class output)
Cheng et al.^43^ (2020)	HNN	MRI	Sagittal and 3D reconstruction	Manual segmentation, >15 yr of experience	80	9-Fold cross	10	Probability maps for clinical conditions
Hu et al.^44^ (2024)	TRGCN	MRI	2D and 3D	MSK radiologist segmentations	155 OA 325 Control	Not specified	39 OA 81 Control	Simulated PF tracking

AI, artificial intelligence; CNN, convolutional neural network; CT, computed tomography; D-CNN, dilated convolutional neural network; GBM, gradient boosting machine; GBM-CNN, gradient boosting machine and convolutional neural network; HNN, hypercomplex neural network; HRNet, high-resolution network; KL, Kellgren-Lawrence; MI-CNN, multi-instance convolutional neural network; MRI, magnetic resonance imaging; MSK, musculoskeletal; MWRN, multi-wavelet residual network; OA, osteoarthritis; OARSI, Osteoarthritis Research Society International; PF, patellofemoral; PFI, patellofemoral insufficiency; PFPS, patellofemoral pain syndrome; R-CNN, region-based convolutional neural network; ResNet, residual network; SSPA-NN–SSM, supervised spatiotemporal aggregation neural network and spatial structure mining; SVM, support vector machine; TRGCN, temporal relational graph convolutional network; 2D, 2-dimensional; 3D, 3-dimensional.

#### Overview of Common Convolutional Neural Network Architectures Used in PF Pathology Studies

ResNet and U-Net convolutional neural networks (CNNs) are among the widely used CNN architectures. ResNet addresses the vanishing gradient problem by introducing skip connections, allowing for the training of much deeper networks. ResNet-50 and ResNet-101 are notable examples that have achieved significant success in image classification and other computer vision tasks. U-Net, primarily used for biomedical image segmentation, features a contracting path to capture context and a symmetrical expanding path for precise localization. It is particularly effective with small training datasets. High-resolution networks (HRNet) and multi-instance convolutional neural networks (MI-CNN) further extend CNN capabilities. HRNet maintains high-resolution representations throughout the network, which are crucial for tasks requiring high spatial precision, such as pose estimation and object detection. MI-CNN is designed to handle multiple instances within an image, making it suitable for detecting multiple objects or regions of interest.

##### Object Detection and Segmentation Models

Region-based convolutional neural networks (R-CNN) and dilated convolutional neural networks (D-CNN) are pivotal in object detection and semantic segmentation. R-CNN extracts region proposals and applies a CNN to each region, a method enhanced by its variants, Fast R-CNN and Faster R-CNN, for improved efficiency and accuracy. D-CNN uses dilated convolutions to expand the receptive field without increasing the number of parameters, effectively capturing context at multiple scales, which is essential for tasks such as semantic segmentation.

##### Hybrid and Specialized Models

The gradient boosting machine and convolutional neural network (GBM-CNN) model combines the strengths of CNNs for initial image processing and GBMs for final predictions. This hybrid model can significantly enhance performance in diverse implementations. The supervised spatiotemporal aggregation neural network and spatial structure mining (SSPA-NN–SSM) model leverages a combined approach to aggregate spatiotemporal information and extract spatial structures, which is beneficial for time-series data or spatial dependencies.

##### Advanced Neural Networks

Multi-wavelet residual networks (MWRN) integrate wavelet transforms with residual networks to capture both spatial and frequency information, making them useful for multiscale feature analysis. Hypercomplex neural networks (HNN) extend traditional neural networks to handle hypercomplex numbers, enabling efficient processing of multidimensional data, which is advantageous in areas such as signal processing and 3-dimensional (3D) graphics.

##### Temporal and Graph-based Models

Temporal relational graph convolutional networks (TRGCN) and support vector machines (SVM) illustrate the application of graph-based and traditional ML models in conjunction with DL. TRGCN combines graph convolutional networks with temporal information, which is suitable for analyzing structured data that evolve over time, such as social networks or video data. Although not a DL model, SVM is often integrated with CNNs to enhance classification performance, especially in scenarios with limited datasets.

##### Image Input, Image Planes, and Ground Truth

The aforementioned models use a variety of imaging modalities, including CT scans, MRI scans, and radiographs, across different planes (axial, sagittal, coronal, dynamic, and 3D reconstructions). For example, ResNet and SSPA-NN–SSM use CT scans with ground truth provided by MSK radiologists and previous algorithm segmentations, respectively. Models such as GBM-CNN, MI-CNN, HRNet, and R-CNN primarily use radiographs validated through various standards, such as comparisons of multiple algorithms, single-input CNNs, MSK radiologists, and independent expert graders. MRI scans are used by MWRN, U-Net CNN, D-CNN, SVM, HNN, and TRGCN, with ground truth provided by arthroscopy, senior surgeons, extensive databases, and radiologists with significant experience.

### AI Model Results

Overall, grouped models such as ResNet and SSPA-NN–SSM used CT scan inputs. ResNet focused specifically on axial planes and was validated by MSK radiologists. In contrast, SSPA-NN–SSM covered sagittal, coronal, axial, and 3D reconstructions, relying on ground truth from prior algorithm outputs or manual segmentations. Other models, such as GBM-CNN, MI-CNN, HRNet, and R-CNN, primarily used radiographs for model input. The GBM-CNN model focused on sagittal planes and used a comparison of multiple algorithms for validation. MI-CNN modeling used dynamic radiographs validated by a single-input CNN. The HRNet system relied on axial radiographs with validation from 2 MSK radiologists, and R-CNN used sagittal radiographs assessed by 2 independent expert Osteoarthritis Research Society International (OARSI) graders. Several models, including MWRN, U-Net CNN, D-CNN, SVM, HNN, and TRGCN, used MRI scans. MWRN used sagittal, coronal, and axial planes with ground truth provided by arthroscopy. U-Net CNN models, which also used CT scans, spanned multiple planes (sagittal, coronal, axial, and 3D reconstructions) and were validated by various standards, including MSK physician experts and previous algorithm segmentations. D-CNN used a combination of MRI scans and radiographs, covering multiple planes and 3D reconstructions, with validation from sources such as the Biomarker Consortium Database and independent radiologists. SVM used MRI scans across sagittal, coronal, and axial planes, relying on radiologists with over 5 years of experience. HNN used sagittal and 3D reconstructions with manual segmentation expertise spanning more than 15 years. TRGCN integrated both 2-dimensional and 3D MRI planes, validated by MSK radiologist segmentations. Overall, the ground truth/reference standards for these models varied widely. These standards included evaluations by MSK radiologists, senior surgeons, and arthroscopy; comparisons with multiple algorithms; and evaluations using large databases.

### AI Model Performance, Predictive Capacity, and Quantitative Metrics for PF Pathology

**Fig 2:**
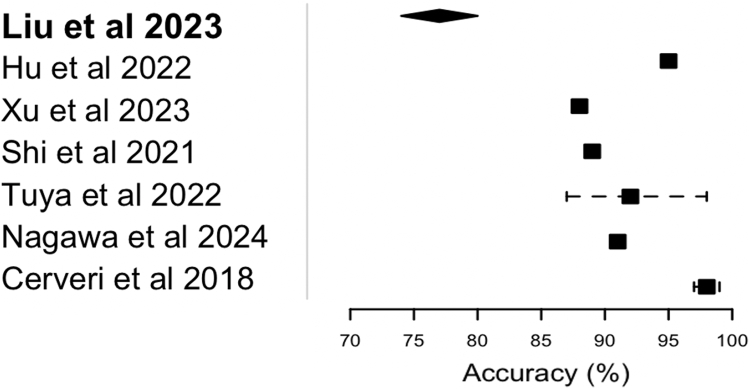
Forest plot comparing performance accuracy for eligible artificial intelligence models (n = 7). Diamond shape and dashed lines represent range bars of accuracy % reported in the respective study. Squares are the shape designated for standard representation of accuracy.

**Fig 3:**
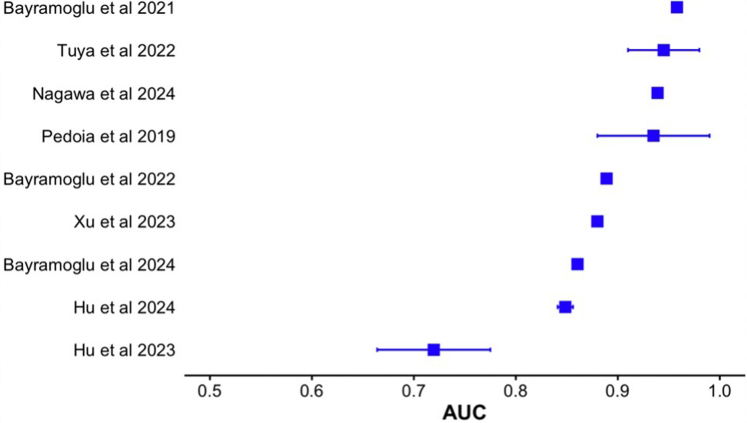
Forest plot comparing area under curve (AUC) for eligible artificial intelligence models (n = 9). Dashed lines represent range bars of accuracy % reported in the respective study. Squares are the shape designated for standard representation of accuracy.

In evaluating the performance metrics across the included studies, distinct trends and differences were described and grouped based on performance and predictive capacity for accuracy, AUC, AP, dispersion of data (MAE, MAD, and RMS), inter-rater reliability (κ value or ICC), sensitivity, specificity, PPV, NPV, and Dice coefficient (Table 3). The most standardized metrics in the literature for quantitative assessment of model performance, study prediction accuracy, and AUC are represented in Figures 2 and 3.

**Table 3:** Overview of Performance Metrics for AI Models in Across Studies

Study	Accuracy	AUC	AP	Dispersion of Data (MAE, MAD, and/or RMS)	κ Value or ICC	Sensitivity	Specificity	PPV	NPV	Dice Coefficient	Sample Size
Liu et al.^29^ (2023)	0.74-0.80	Not included	Not included	0.20-0.26	Not included	Not included	Not included	Not included	Not included	Not included	483
Bayramoglu et al.^30^ (2022)	Not included	0.889	0.714	Not included	Not included	Not included	Not included	Not included	Not included	Not included	5,507
Hu et al.^31^ (2022)	0.95	Not included	Not included	Not included	κ: 0.682-0.748	Not included	Not included	Not included	Not included	Not included	104
Xu et al.^32^ (2023)	0.88	0.88	Not included	Not included	1	0.79	0.96	0.94	0.84	Not included	464
Shi et al.^33^ (2021)	0.89	Not included	Not included	Not included	Not included	0.976	0.76	Not included	Not included	Not included	41
Yurova et al.^34^ (2024)	Not included	Not included	Not included	Not included	Not included	Not included	Not included	Not included	Not included	0.9838	15
Tuya et al.^35^ (2023)	0.87-0.98	0.91-0.98	Not included	Not included	Not included	0.75-0.87	0.9-0.99	0.69-0.9	0.92-0.99	Not included	1,280
Tuya et al.^36^ (2023)	Not included	Not included	Not included	0.06-5.09	ICC: 0.85-0.97	Not included	Not included	Not included	Not included	Not included	1,431
Bayramoglu et al.^22^ (2021)	Not included	0.958	0.862	Not included	Not included	Not included	Not included	Not included	Not included	Not included	2,803
Hu et al.^37^ (2023)	Not included	0.664-0.775		Not included	Not included	Not included	Not included	Not included	Not included	Not included	364
Bayramoglu et al.^38^ (2024)	Not included	0.856-0.865	0.431-0.447	Not included	Not included	Not included	Not included	Not included	Not included	Not included	1,832
Nagawa et al.^39^ (2024)	0.909	0.939	Not included	Not included	Not included	Not included	Not included	Not included	Not included	Not included	49
Barbosa et al.^40^ (2024)	Not included	Not included	Not included	1.38 ± 0.76	ICC: 0.75-0.9	Not included	Not included	Not included	Not included	Not included	95
Cerveri et al.^41^ (2018)	0.97-0.99	Not included	Not included	Not included	Not included	Not included	Not included	Not included	Not included	Not included	232
Pedoia et al.^42^ (2019)	Not included	0.88-0.99	Not included	Not included	Not included	Not included	Not included	Not included	Not included	Not included	302
Cheng et al.^43^ (2020)	Not included	Not included	Not included	0.41-0.44	Not included	Not included	Not included	Not included	Not included	0.94-0.97	93
Hu et al.^44^ (2024)	Not included	0.841-0.856	Not included	Not included	Not included	Not included	Not included	Not included	Not included	Not included	600

NOTE. Data are presented as mean ± standard deviation unless otherwise indicated.

AI, artificial intelligence; AP, average precision; AUC, area under curve; ICC, intraclass correlation coefficient; MAD, mean absolute deviation; MAE, mean absolute error; NPV, negative predictive value; PPV, positive predictive value; RMS, root-mean-square.

##### Superior-Performance Studies

Superior-performance studies showed the highest levels of accuracy, AUC, and additional performance metrics, often supported by robust sample sizes and comprehensive methodologies. Clinically, these studies evaluated the AI models’ ability to identify specific PF abnormalities such as trochlear dysplasia, OA severity, and patellar instability using imaging biomarkers. For example, Cerveri et al.^41^ (2018) reported an excellent accuracy range of 0.97 to 0.99, with a sample size of 232, for staging of distal femoral trochlear dysplasia. Similarly, Tuya et al.^35^ (2023) achieved an accuracy range of 0.87 to 0.98 and an AUC between 0.91 to 0.98, with sensitivity values ranging from 0.9 to 0.99 and specificity between 0.69 and 0.9, with a large sample size of 1,280, for diagnosis and grading of PF OA. Additionally, Xu et al.^32^ (2023) reported an accuracy of 0.88 and an AUC of 0.88, alongside a sensitivity of 0.79 and specificity of 0.96, for diagnosis of femoral trochlear dysplasia based on MRI measurements. The study also noted a κ value of 1, a PPV of 0.94, and an NPV of 0.84, with a sample size of 464. Finally, Cheng et al.^43^ (2020) reported dispersion data with a very low margin of error (0.41-0.44) and a Dice coefficient of 0.94 to 0.97, based on a sample size of 93, for PF MRI segmentation.

##### High-Performance Studies

There were also studies that exhibited strong, though slightly lower, performance metrics compared with the superior group. Bayramoglu et al.^30^ (2022) reported an AUC of 0.889 and an AP of 0.714, with a large sample size of 5,507, for predicting PF OA progression from initial baseline imaging features. Hu et al.^31^ (2022) showed an accuracy of 0.95 and a κ range of 0.682 to 0.748, with a smaller sample size of 104, for diagnosing initial cartilage lesions on baseline MRI and then predicting eventual progression of OA based on degree of initial injury. Shi et al.^33^ (2021) reported an accuracy of 0.89 and an ICC of 0.976, with a sample size of 41, for diagnosis of PF pain syndrome. Finally, the study by Bayramoglu et al.^22^ (2021) qualified as a high-performance study by achieving an AUC of 0.958 and an AP of 0.862, with a large sample size of 2,803, for detection of PF OA from knee lateral-view radiographs.

##### Moderate-Performance Studies

Moderate-level studies presented reasonable but less consistent results, often accompanied by a range of performance metrics and varying sample sizes. Tuya et al.^36^ (2023) reported dispersion data (MAE, MAD, and/or RMS) ranging from 0.06 to 5.09 and an ICC of 0.85 to 0.97, with a sample size of 1,431. Similarly, Hu et al.^37^ (2023) reported an AUC range of 0.664 to 0.775, with a sample size of 364, but did not report any additional outcome metrics. Last, Liu et al.^29^ (2023) reported a low dispersion of data in their results, with a moderate sample size (n = 483) and suboptimal accuracy (0.74-0.8).

##### Poor-Performance Studies

Several studies either scored low in performance metrics or had favorable metrics but presented incomplete data or limited outputs. Bayramoglu et al.^38^ (2024) reported an AUC range of 0.856 to 0.865 and an AP range of 0.431 to 0.447, with a sample size of 1,832. Similarly, Nagawa et al.^39^ (2024) showed an accuracy of 0.909 and an AUC of 0.939 but with a small sample size of 49. Barbosa et al.^40^ (2024) provided dispersion data (RMS, 1.38 ± 0.76) and a κ range of 0.75 to 0.9, with a sample size of 95. Hu et al.^44^ (2024) had an AUC range of 0.841 to 0.856, with a sample size of 600, but did not report on any additional metrics for evaluation of model output. Yurova et al.^34^ (2024) reported a Dice coefficient of 0.9838, with a limited sample size of 15. Finally, Pedoia et al.^42^ (2019) had a large sample size (n = 302) and an excellent model AUC value ranging from 0.88 to 0.99 but failed to report more than one outcome metric.

### Quality Assessment

Quality assessment was conducted using the modified MINORS score, which has been previously established in the literature for evaluating AI models.^20^ The average MINORS score among the included studies was 5.35 ± 0.68, indicating a high level of overall methodologic quality. However, the analysis revealed that most point deductions for methodologic quality evaluation were primarily because of insufficient reporting on dataset distribution. Specifically, many studies failed to adequately describe the parameters of the training, validation, and testing phases of their datasets. Additionally, another area of weakness was the failure to clearly define a ground truth for reference AI standards. This is crucial for accurately assessing the performance and reliability of AI models to serve as a benchmark against which the model’s outputs can be compared. For example, two studies focused primarily on developing proof-of-concept models without external validation or comprehensive data splits, limiting their generalizability and increasing the risk of overfitting.^32^^,^^35^ These methodologic shortcomings reduce confidence in the robustness of their findings and highlight the need for clearer validation protocols. As highlighted by Kunze et al.,^20^ these internal study design weaknesses introduce a significant risk of publication bias, which arises when inadequately validated AI models are published and accepted into the scientific literature without sufficient scrutiny. Consequently, these poorly trained models can persist and potentially mislead future research and applications in the field.

## Discussion

The primary finding of this study was that the AUC and prediction accuracy of AI models for diagnosing and predicting PF pathology ranged from 0.664 to 0.990 and 74% to 99%, respectively. However, there was significant heterogeneity in methodology and outcome reporting in the studies analyzed, which makes generalizability across studies challenging but provides several avenues for further model refinement. The use of AI in diagnosing PF compartment pathology has emerged as an important development in orthopaedic medicine.^42^ This study offers a detailed evaluation of several AI models, examining their diagnostic accuracy, predictive capabilities, and overall performance.

The AI models reviewed in this study exhibit a broad spectrum of diagnostic capabilities, demonstrating their potential to enhance clinical practice. Notably, the models developed by Cerveri et al.^41^ (2018) and Tuya et al.^35^ (2023) achieved exceptional and near-perfect diagnostic accuracy, with accuracy rates reaching 0.97 to 0.99 and AUC values ranging from 0.91 to 0.98, respectively. These high metrics indicate that these models are well suited for identifying PF compartment pathology, potentially leading to improved diagnostic precision and patient outcomes. Similarly, the models of Yurova et al.^34^ (2024) and Cheng et al.^43^ (2020) stand out for their robust performance in segmentation tasks, as evidenced by high Dice coefficients of 0.984 and 0.94 to 0.94, respectively. The Dice coefficient, which measures the overlap between the predicted and actual segmentation results, reflects the model’s ability to accurately delineate PF compartment structures such as the trochlear groove and patellar facets—a critical component for effective diagnosis and treatment planning. Additionally, Xu et al.^32^ (2023) reported an accuracy of 0.88 and an AUC of 0.88, alongside a sensitivity of 0.79 and specificity of 0.96. The study also noted a κ value of 1, a PPV of 0.94, and an NPV of 0.84, with a sample size of 464.^32^ The comprehensive reporting of multiple performance metrics in the study by Xu et al. illustrates the multifaceted approach needed to fully assess model effectiveness. For the additional models evaluated in the study, incomplete outcome metric reporting dampens the ability to draw definitive conclusions about model performance and highlights the necessity for uniformity across AI model studies on PF pathology.

Although most studies centered on identifying and diagnosing PF pathology, several progressed to the next logical step of predicting disease progression and clinical outcomes based on initial imaging biomarkers. For example, Bayramoglu et al.^30^ (2022) showed the ability to predict the progression of OA from baseline patellar bone texture features on radiographs. Similarly, Hu et al.^44^ (2024) developed a model that learned the intrinsic connections and longitudinal patterns of knee structures and compartments to provide interpretable insights for identifying and predicting knee OA progression. Finally, Nagawa et al.^39^ (2024) developed an ML-based prediction model for PF instability using 3D MRI-based shape models of femurs and obtained favorable predictive performance. These studies suggest that the future of AI in clinical practice could evolve from being used solely for diagnostic purposes to also incorporating predictive capabilities. By using radiographic or cross-sectional imaging—which is already a standard-of-care order in the clinical pathway—to analyze anatomic features, AI could assist clinicians in predicting disease patterns, PF instability, and progression of OA.

For quality assessment and methodologic considerations, the application of the modified MINORS score revealed an average score of 5.35 ± 0.68 across the studies, reflecting generally high methodologic quality. This score indicates that most studies adhered to rigorous standards in AI model development and evaluation. However, several studies did not provide detailed information regarding dataset distribution, sample size, or ground-truth definitions. Such omissions can introduce biases and affect the reliability of performance metrics. Comprehensive reporting is crucial for the accurate assessment of AI models. Details on dataset composition, model training processes, and validation techniques are essential for understanding model performance and ensuring reproducibility. The lack of such information in some studies points to a broader need for standardized reporting practices in AI research to enhance the clarity and reliability of findings.^45^, ^46^, ^47^ As discussed earlier, the current limitations in AI applications for the diagnosis of PF pathology will likely serve as the catalyst for model refinement and the development of standardized guidelines for reliable and reproducible DL and ML research.

Recently, several studies have highlighted the excellent diagnostic performance of AI for various orthopaedic pathologies.^7^^,^^20^^,^^48^, ^49^, ^50^ For example, Kunze et al.^20^ analyzed the diagnostic efficacy of AI methods for detecting anterior cruciate ligament and meniscus tears and found that although AI prediction and performance were excellent, AI models did not outperform clinical experts. They concluded that AI should be used as an adjunctive tool to enhance the diagnostic capabilities of human experts. Similarly, Ashinsky et al.^21^ (2017) used ML to identify MRI biomarkers from cartilage mapping of the femoral condyle to predict early OA progression with a high degree of sensitivity and accuracy. Finally, de Carvalho et al.^51^ (2022) developed an advanced, semi-automated segmentation model for the diagnosis of hallux rigidus that reliably expressed excellent interobserver and intraobserver reliability with a high degree of reproducibility compared with ground-truth manual segmentation. These studies highlight significant implications for the future of diagnostics in orthopaedic sports medicine, urging physicians to become proficient with this technology as the era of digital consumerism and innovation continues to expand.

In terms of clinical relevance, the potential clinical impact of high-performing AI models is substantial. These models can enhance diagnostic precision and efficiency for PF compartment pathologies while reducing health care costs. High-performance AI tools have the potential not only to improve diagnostic accuracy but also to facilitate early identification and evidence-based clinical decision making. One particularly promising aspect of AI is its ability to predict disease progression from anatomic features using baseline data input (radiographs, MRI scans, and so on). Predictive models leverage ML algorithms to analyze these data and forecast how PF pathologies may develop over time. This includes anticipating changes in disease severity, response to treatment, and long-term outcomes. Such predictive capabilities would enable more personalized and proactive treatment strategies, potentially leading to better management of the disease. Although this field is still evolving, integrating AI into clinical practice has the potential to streamline diagnostic workflows, reduce errors, and enhance patient outcomes.

Despite the promising results, several improvements are required in the AI model sphere to enhance the applicability of AI models in orthopaedic surgery for ubiquitous use by clinicians. The variance and non-uniformity in performance metrics and their reporting highlight the need for ongoing refinement and calibration of AI models. Moreover, the variability in dataset sizes and quality across studies suggests that larger, more diverse datasets are necessary to improve model generalizability. Our study findings emphasize the importance of including a broad range of patient demographic variables and clinical scenarios to enhance the robustness and applicability of AI models. Future research should focus on several key areas: standardizing reporting practices to ensure transparency and reproducibility, conducting multicenter validation studies to assess AI models in varied clinical environments, and exploring the integration of AI with other diagnostic tools and clinical data.

### Limitations

Several limitations should be noted. The screening and selection process was restricted to articles meeting strict inclusion and exclusion criteria, which could have led to the exclusion of relevant studies that did not conform to these criteria. The heterogeneity in study designs, sample sizes, imaging parameters, inclusion and exclusion criteria, and methodologies limits the applicability of the findings to clinical practice. Moreover, the reliance on qualitative synthesis and summary statistics may obscure more nuanced results and trends. Finally, although this study adhered to the PRISMA guidelines as a systematic review, a quantitative meta-analysis could not be performed because of limited data and study heterogeneity. This constraint impacts the robustness of the conclusions that can be drawn from our study.

## Conclusions

AI models showed variable diagnostic performance in identifying PF pathologies and predicting disease progression, with reported AUCs ranging from 0.664 to 0.990 and accuracies between 74% and 99%. Although some studies suggested that AI outperformed traditional diagnostic methods such as interpretation by MSK radiologists, manual segmentation, or arthroscopy, the degree of superiority was inconsistent and influenced by significant heterogeneity in model architectures, imaging modalities, and reference standards. Given the broad scope of this review and variability across studies, caution is warranted in interpreting these findings, and specific clinical recommendations cannot be made at this time.

## Disclosures

All authors (J.T-K., M.A.B., Z.W.H., S.L., L.M., A.M., E.H., D.V.B., K.B., J.W., B.L.) declare that they have no known competing financial interests or personal relationships that could have appeared to influence the work reported in this paper.
